# Synthesis and spectroscopic properties of new bis-tetrazoles

**DOI:** 10.1007/s10847-012-0219-4

**Published:** 2012-07-22

**Authors:** Agnieszka Pazik, Anna Skwierawska

**Affiliations:** Department of Chemical Technology, Chemical Faculty, Gdansk University of Technology, 80-233 Gdańsk, Poland

**Keywords:** Tetrazole, X-ray, IR, UV–Vis

## Abstract

Syntheses of *N,N*′-phenyltetrazole podands link with aliphatic chains containing oxygen, nitrogen and sulphur atoms, are described. The complexing properties of these compounds towards metal cations (Fe^2+^, Cu^2+^, Zn^2+^, Co^2+^, Ni^2+^) were investigated by absorption and infrared spectroscopy. The UV–Vis titrations were performed to estimate the stability constant values of the respective complexes with Cu^2+^ ion. Changes in UV–Vis absorption spectra and IR spectra of compound **6** under various concentrations of Cu^2+^ ion in methanol suggest formation of very unstable complex. The structure of ligand **2** has been deduced by X-ray crystallography.

## Introduction

Numerous proposals have been made over a considerable period for the production of the synthetic tetrazoles from nitriles, amines and amides. The tetrazole function is metabolically stable and this feature and a close resemblance between the acidic character of the tetrazole group and the carboxylic group has inspired syntheses of potential therapeutic agents. Due to the possibility of deprotonation, protonation and alkylation amongst other reactions, the properties of tetrazoles can be considerably varied and result in numerous possible derivatives. The tetrazole ring is resistant to the action of acids, bases and reducing agents. Tetrazolate anions have dual reactivity, form stable complexes with metals and halogens. They have wide applications as corrosion inhibitors [1], analytical reagents, high-energy materials [2, 3] and component of ionic liquid and gas generating compositions [4]. Tetrazoles play important role in coordination chemistry as ligands [5], in medicinal chemistry as metabolically stable substitutes for carboxylic acids. However, tetrazoles themselves exhibit no pharmacological activity; many of their derivatives possess interesting biological (anti-hypertensive, anti-allergic and antibiotic) activities [6]. In recent years, particular attention has been directed to mono- or bidentate tetrazole ligands as molecular hosts in generating supramolecular arrays and functionalized poly-tetrazoles such as sensors.

Methods of synthesis monosubstituted tetrazoles, which include 1-, 2- and 5-monosubstituted tetrazoles, were the main subject of various studies. The choice of synthetic methods of 1-substituted tetrazoles is limited. One of the most known methods of synthesis 1-substituted tetrazoles is cycloaddition of isocyanides to hydrazoic acid. This method was improved replacing hydrazoic acid by a more effective reagent, trimethylsilyl azide (Me_3_SiN_3_) [7]. Another method involves the reaction of amines with ethyl orthoformate and sodium azide in glacial acetic acid. Aromatic, aliphatic and heteroaromatic amines can be used for preparation of tetrazoles and bis-tetrazole derivatives. 2-Substituted tetrazoles are usually synthesized by alkylation or acylation of tetrazole and that reactions are characterized by low selectivity and poor yield. 5-Substituted tetrazoles can be prepared in several ways; the commonest method of synthesis involves the cycloaddition of nitriles to azides. The reaction is general and is widely used in the synthesis of tetrazoles derivatives in the design of new drugs containing a tetrazole ring. Nitriles possessing various functional groups and dinitriles can be used as initial compounds. Interesting is synthesis of substituted tetrazoles in multistep reaction of aldehydes with iodine in aqueous ammonia, followed by addition of sodium azide in the presence of Lewis acids (e.g., ZnCl_2_ or ZnBr_2_) [8] in the mixture of water, ammonium chloride and *N,N*-dimethylformamide. In classical synthesis of tetrazoles, hydrazoic acid or sodium azide reacts with imidoyl chloride intermediate which is formed in reaction of amide with phosphorus(V) chloride [9]. Noteworthy is one-step conversion of secondary monoamides to tetrazoles under Mitsunobu reaction conditions with azides, especially with tributyl- or trimethylsilyl azide which are safe and soluble in organic solvents [10, 11]. An alternate method employs trifluoromethanesulfonic anhydride and sodium azide for imidoyl derivatives preparation, and thus is converted to tetrazoles [12]. It is commonly known that application of this methodology to the bisamides is impractical because of poor yield and low solubility of this type of compounds in the typical Mitsunobu reaction medium (e.g., anhydrous THF or dichloromethane).

**Fig. 1:**
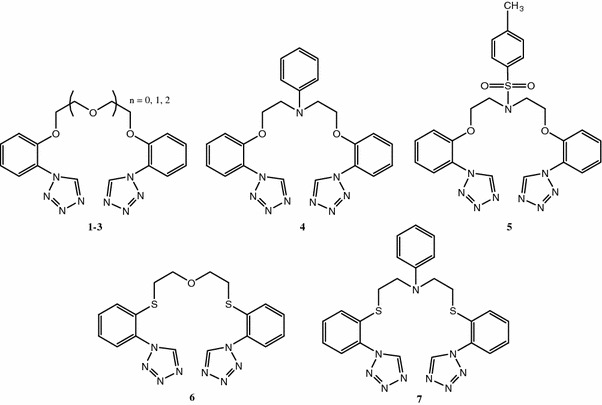
General formula of the macrocycles

The purpose of this work was synthesis of new bis-tetrazoles containing chains with different heteroatoms. The structures of new compounds are presented in Fig. 1. Influence of ligands chemical structure on cation metal complexation was studied by means of IR and UV–Vis spectroscopy.

## Results and discussion

### Synthesis

**Scheme 1:**
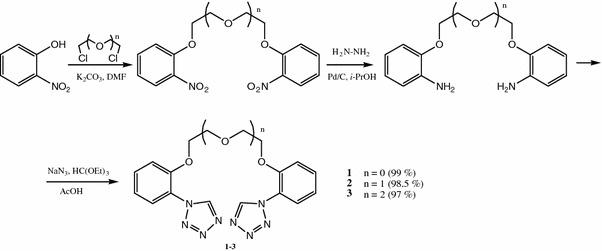
Synthesis of bis-[2-(1*H*-tetrazo-1-yl)phenoxy]-ethane derivatives

Bis-tetrazole compounds **1**–**7** which possess in their structure aromatic rings linked by chains with heteroatoms, such as oxygen, nitrogen and sulphur were synthesized. Compounds **1**–**3** were obtained in three-stage reaction: alkylation of 2-nitrophenol, reduction of bis-nitroderivatives follow by heterocyclic ring formation (Scheme 1).

**Scheme 2:**
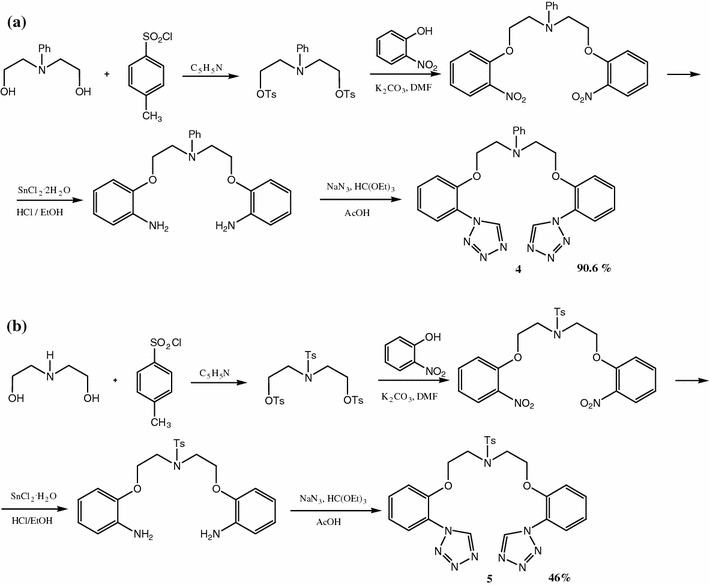
Synthesis of bis-[2-(1*H*-tetrazo-1-yl)phenoxy)-3-azapentane derivatives

The same procedure was applied in synthesis of compounds **4** and **5**, but well established reduction with hydrazine at presence of Pd/C catalyst was found ineffective and SnCl_2_ in hydrochloric acid solution was used (Scheme 2).

**Scheme 3:**
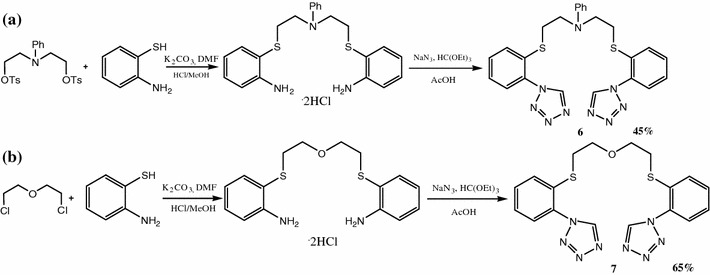
**a** Synthesis of bis-[2-(1*H*-tetrazo-1-yl)phenyl(thio)]-3-phenylazapentane, **b** synthesis of bis-[2-(1*H*-tetrazo-1-yl)phenyl(thio)]-3-oxapentane

Ligands contain sulphur atoms were obtained in two step reactions: selective alkylation of 2-aminotiobenzene and amine groups transformation into tetrazole rings (see Scheme 3).

In all cases tetrazole residues were obtained in reaction of amines with ethyl orthoformate and sodium azide in glacial acetic acid. The reaction occurs under mild conditions and the yield of products is very high. The best yield over 97 % was obtained in case of compounds **1**–**3**. Final products (**1**–**5**) were purified by crystallization. The compounds are white (**1**–**3**) or brown (**4**) solids. The oil compounds **6**–**7** were purified by column chromatography.

**Fig. 2:**
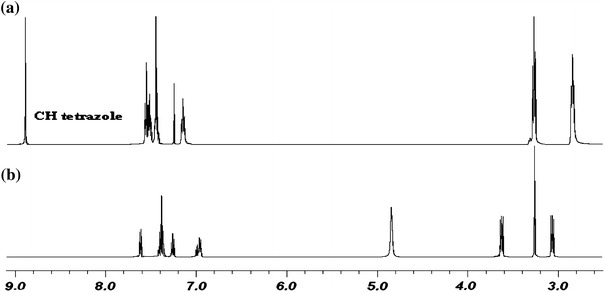
**a** ^1^H NMR spectra of **6** in CDCl_3_, **b** ^1^H NMR spectra of 1,5-bis[2-aminophenyl(thio)]-3-phenylazapentane hydrochlochloride in d-MeOH

The infrared spectroscopy is excellent technique to study the progress of cyclization reaction. IR Spectra of reagents show typical amine and azide bands at ca. 3400 and ca. 2060 cm^−1^, respectively, which are decreased adequately to tetrazole rings formation. It is worth to mention that in tetrazoles’ ^1^H NMR spectra characteristic C–H signal at 8.99–9.77 ppm is observed. As a comparison, two ^1^H NMR spectra of 1,5-bis[2-aminophenyl(thio)]-3-phenylazapentane and 1,5-bis[2-(1*H*-tetrazol-1-yl)phenyl(thio)]-3-phenylazapentane (**6**) are presented in a Fig. 2. Singlet at 8.90 ppm corresponds to two C–H tetrazole protons of compound **6**.

### X-ray structural analysis of the 1,5-bis[2-(1*H*-tetrazol-1-yl)phenoxy]-3-oxapentane (**2**)

Experimental data were collected on the KM4 κ-geometry diffractometer equipped with Sapphire 2 CCD detector (Oxford Diffraction). Enhanced Mo K_α1_ X-ray radiation source with a graphite monochromator was used. Measurements were carried out in four ω-scan runs—scan width 0.75º, exposure time 300 s per frame was rather long due to weak diffracting power of all the specimen. Determination of the unit cell and data collection was carried out at room temperature, i.e., 293(2) K. All preliminary calculations were performed using CrysAlis RED and CrysAlis CCD software package (Oxford Diffraction, 2010).

The structure was solved by direct methods. Refinement was made against all reflections by the full-matrix least squares procedure based on *F* ^2^. All of the non-hydrogen atoms were refined with anisotropic thermal parameters. Hydrogen atoms were refined as riding with isotropic *U* values fixed as 1.2 times *U*eq of the respective pivot atom. Due to low value of absorption no correction was applied. The calculations were carried out using the SHELX-97 (G. Sheldrick, 1997) program package, run under WinGX 1.70.00 (L. Farrugia, 1999) Windows shell program.

**Fig. 3:**
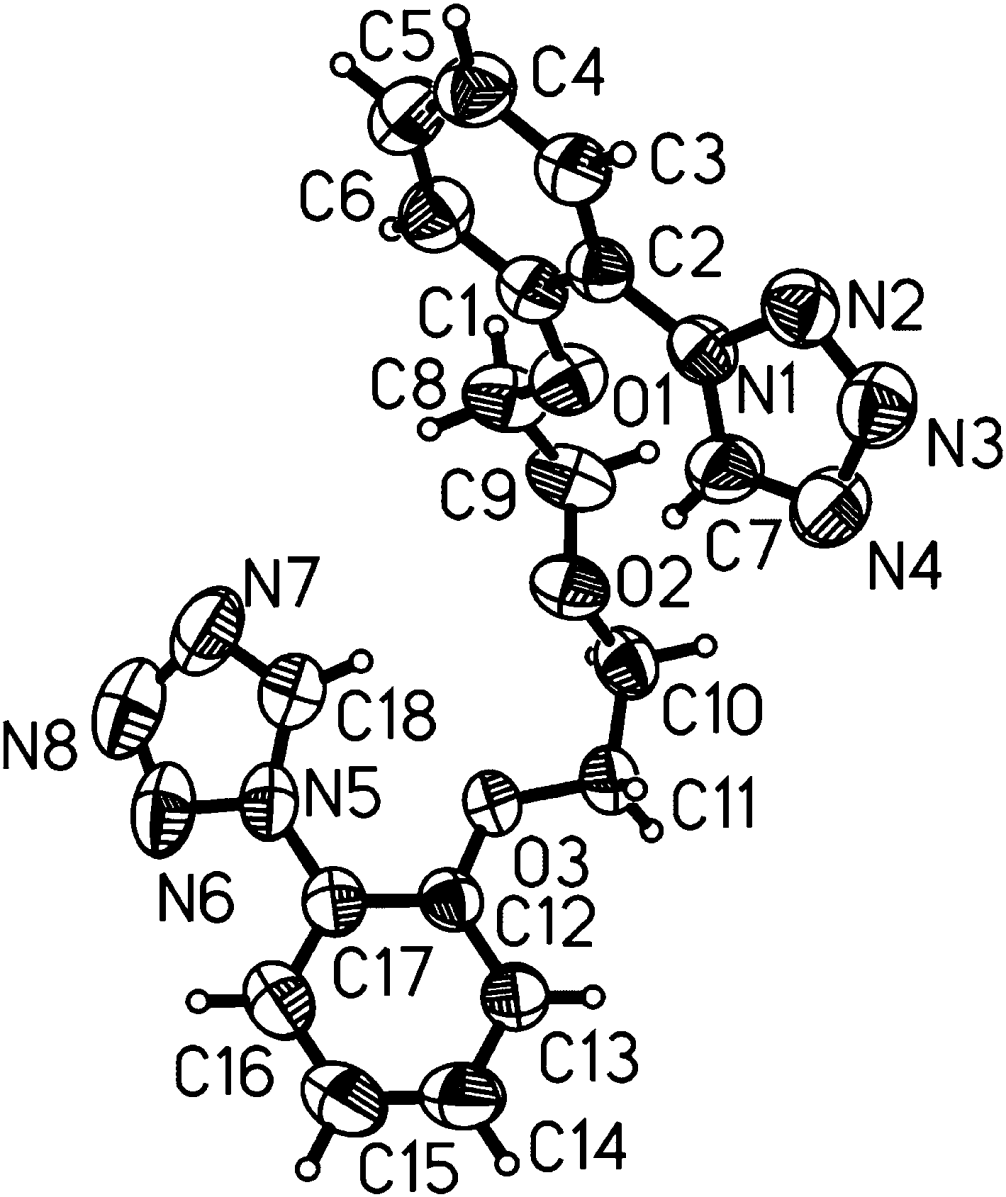
Molecular structure of **2**, displacement *ellipsoids* drawn at 50 % probability level

Compound **2** crystallizes in monoclinic space group *P*2_1_/*c* with four molecules in the unit cell. Molecular structure together with the atom labeling scheme is shown in Fig. 3. The system of rings is not flat. Dihedral angle between mean planes defined by skeletal atoms of C1–C6 phenyl ring and N1–N4–C7 tetrazole ring is equal to 24.88º, and between C12 and C17 phenyl ring and N5–N8–C18 tetrazole ring is even wider 36.03º.

**Fig. 4:**
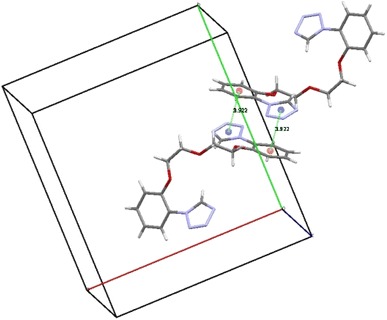
Stacking interactions in crystals off compound **2** in vicinity of inversion centre

No classical hydrogen bonds can be found in the structure of podand **2**. Only one stronger stacking interaction can be found in the structure, namely between N1–N4–C7 tetrazole ring and C1–C6 phenyl ring from the neighbor molecule related by the inversion centre located at (0 1/2 1/2) (symmetry code: −x, 1 − y, 1 − z). The distance of centers of gravity of the two rings is equal to 3.922 Å, all the other rings have centers at mutual distances greater than 4 Å. In fact two of such interactions, related by the inversion center are operating (see Fig. 4). Summary of crystal data and details on data collection of **2** and structure refinement are given in Table 1.

**Table 1:** Crystal data and structure refinement for podand **2**

Compound	**2**
Empirical formula	C_18_H_18_N_8_O_3_
Formula weight	394.40
Temperature	297(2) K
Wavelength	0.71073 Å
Crystal system, space group	Monoclinic, *P*2_1_/*c*
Unit cell dimensions	a = 15.0902(17) Å, α = 90º
	b = 16.7301(18) Å, β = 99.260(10)º
	c = 7.7390(8) Å, γ = 90º
Volume	1928.3(4) Å^3^
Z, calculated density	4, 1.359 Mg/m^3^
Absorption coefficient	0.098 mm^−1^
*F*(000)	824
Crystal size	0.06 × 0.08 × 0.54 mm
θ range for data collection	2.74–26.00º
Limiting indices	−18 ≤ h ≤ 18, −13 ≤ k ≤ 20, −5 ≤ l ≤ 9
Reflections collected/unique	7,039/3,796 [*R*_int_ = 0.0322]
Completeness to θ = 26.00	99.9 %
Refinement method	Full-matrix least squares on *F* ^2^
Data/restraints/parameters	3,796/0/262
Goodness-of-fit on *F* ^2^	0.844
Final *R* indices [*I* > 2σ(I)]	*R*_1_ = 0.0380, *wR*2 = 0.0652
*R* indices (all data)	*R*_1_ = 0.1206, *wR*2 = 0.0786
Largest difference peak and hole	0.115 and −0.111 e A^−3^

### Spectrophotometric studies of the metal cation complexation

In recent years the number of publications and patents describing the structure and physicochemical properties of ligands including in their structure tetrazole heterocyclic fragment has grown intensely. This is due to the wide range of practical applications of these compounds. The high physiological activity and low toxicity of tetrazoles makes it possible to regard their metal complexes as substances of versatile biochemical and pharmaceutical destination. Complex stability depends on ligands structure, essentially position and type of electron donating atoms. Compounds **1**–**3** consist of two 1-*N*-phenyltetrazole residues linked by polyether chain. Ligand **1** in comparison to **2** and **3** forms the most rigged system. More flexible its analogues **1**–**2** have more degrees of freedom during complex formation. In further studies other electron donating atoms such as nitrogen (compounds **4** and **5**) and sulfur (compounds **6** and **7**) were introduced into molecule structure. That modification should improve selective recognition of transition and heavy metal cations.

UV–Vis Absorption spectra of ligands **1**–**7** show three maxima just about 210, 240 and 280 nm in methanol solution. Calculated value of extinction coefficients are summarized in Table 2.

**Table 2:** The absorption maxima (λ_max_) and corresponding extinction coefficients of new bis-tetrazole in MeOH at 298 K

Compound	*λ*_max_ (nm)	*ε* (M^−1^cm^−1^)
1	209	62,000
2	209	59,454
3	210	58,433
6	205	47,520
7	206	58,200

**Fig. 5:**
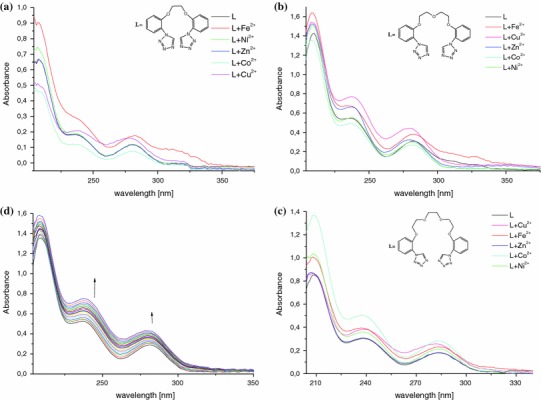
The changes in UV–Vis spectra in the presence of metal chloride (tenfold excess) for compounds **a** **1** (*c* = 2 × 10^−5^ mol dm^−3^); **b** **2** (*c* = 2.5 × 10^−5^ mol dm^−3^); **c 3** (*c* = 2 × 10^−5^ mol dm^−3^) **d** UV–Vis titration of **2** (*c* = 2.5 × 10^−5^ mol dm^−3^) with copper(II) chloride (*c* = 10^−4^ mol dm^−3^, titration step 0.01 ml) in methanol at 298 K

In spectroscopic studies chlorides were used in methanol solution. Any absorbance change in spectra of **1**–**7** was observed in case of Li^+^, Na^+^, K^+^, Sr^2+^, Mg^2+^, and Ca^2+^ chlorides. We expected ligands **1**–**3** response, because of the presence of oxygen atoms which are hard bases according to HSAB theory. In next experiments Fe^2+^, Co^2+^, Zn^2+^, Ni^2+^ and Cu^2+^ chlorides were used. Figure 5 presents spectral absorbance changes of **1**–**3** upon addition of tenfold access of an appropriate salt. Bis-tetrazoles exhibit increase of all maxima upon addition of ferrous(II) chloride. In the presence of copper(II) chloride diminishing of maximum at 210 nm is only observed for **1**. Zinc(II) and nickel(II) chlorides do not influence on ligands absorbance. It is worth to note that only ligand **3** shows positive response to cobalt(II) chloride. In the spectrum all main absorption bands are strengthened. Further spectroscopic titration of compounds **1**–**3** with ferrous(II), copper(II) and cobalt(II) chlorides show continuous increase of the main bands that makes determination of complexation constants impossible. As an example ligand **2** titration with copper(II) solution is shown in the Fig. 5d.

**Fig. 6:**
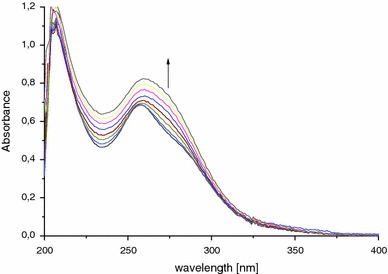
UV–Vis titration of 1,5-bis[2-(1*H*-tetrazol-1-yl)phenyl(thio)]-3-phenylazapentane (*c* = 2.5 × 10^−5^ mol dm^−3^) with copper(II) chloride (*c* = 10^−4^ mol dm^−3^, titration step 0.01 ml)

Very similar pattern of spectroscopic changes in absorption spectra may suggest that polyether chain is not engaged in complex. Another possible explanation is that complex is formed inside of a ligand cavity. Further nitrogen and sulfur atoms were introduce to ligands structure to enhance affinity between ligand and metal cation. This modification do not improved selective recognition of transition or heavy metal cations by ligands. Compounds **4**–**7** were titrated with Fe^2+^, Co^2+^, Zn^2+^, Ni^2+^ and Cu^2+^ chlorides in methanol solution. Very similar results were obtained, with one exception. Compound **6** titrated with copper(II) chloride in methanol solution (Fig. 6), increase in Cu^2+^ concentration caused continuous raise of absorbance maxima at 258 nm. The gradual addition of Cu^2+^ causes also the change of color from colorless to yellow. However after few minutes yellow color disappears that may suggest formation of very unstable complex. Plot of absorbance versus Cu^2+^: **6** ratio showed graduate increase of the absorbance band at 258 nm.

**Fig. 7:**
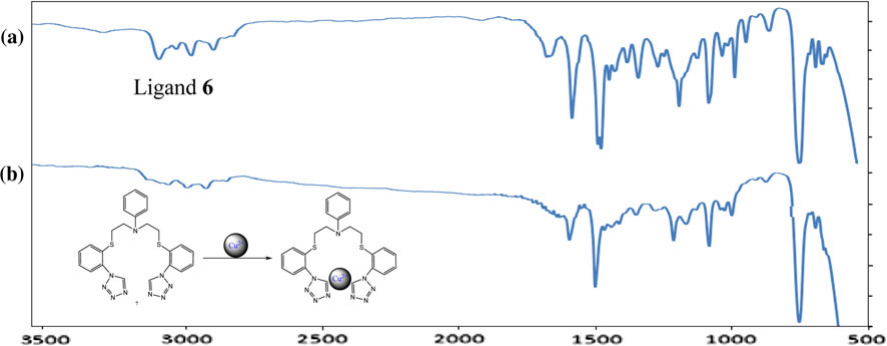
IR spectra (film) of pure ligand **6** (**a**) compared with its complex with copper(II) (**b**)

In order to confirm complexation of **6** with Cu^2+^ ion, series of IR spectra of the ligand **6** in the presence of copper(II) chloride have been recorded within the range of 3500–500 cm^−1^. We took ligand and the copper(II) chloride and dissolved separately in a small amount of chloroform and methanol, respectively. The solutions were combined, stirred, solvent was removed and residue was dried in vacuum. Result solid was dissolved in dry CH_2_Cl_2_ and obtained solution was used for film preparation characterized with IR spectroscopy as shown in Fig. 7.

**Fig. 8:**
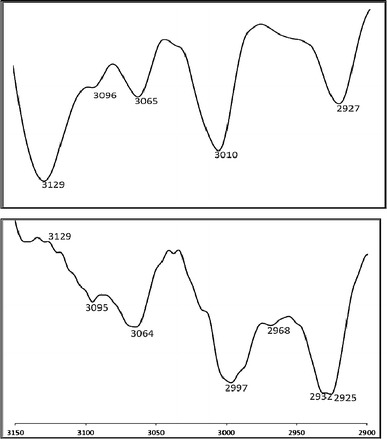
MIR spectra (film) of compound **6** (upper spectrum) and its complex

Figure 7 shows the characteristic down-shifts of the tetrazoles C–H stretching vibration at 3129 cm^−1^ copper(II) coordination. The symmetry of ligand is observed in IR spectroscopy with one large signal deriving from two tetrazoles C–H stretching vibration. A reference spectrum was taken from pure ligand. Figure 8 compares the C–H tetrazole stretching vibrations of pure ligand with the vibrations observed of the new complex in MIR spectroscopy. Complexation can be observed in the loss of stretching vibration at 3129 and the new signals can be found below 3000 cm^−1^. The occurrences of the peaks of the pure ligand at 3065 cm^−1^ prove incomplete complexation.

**Fig. 9:**
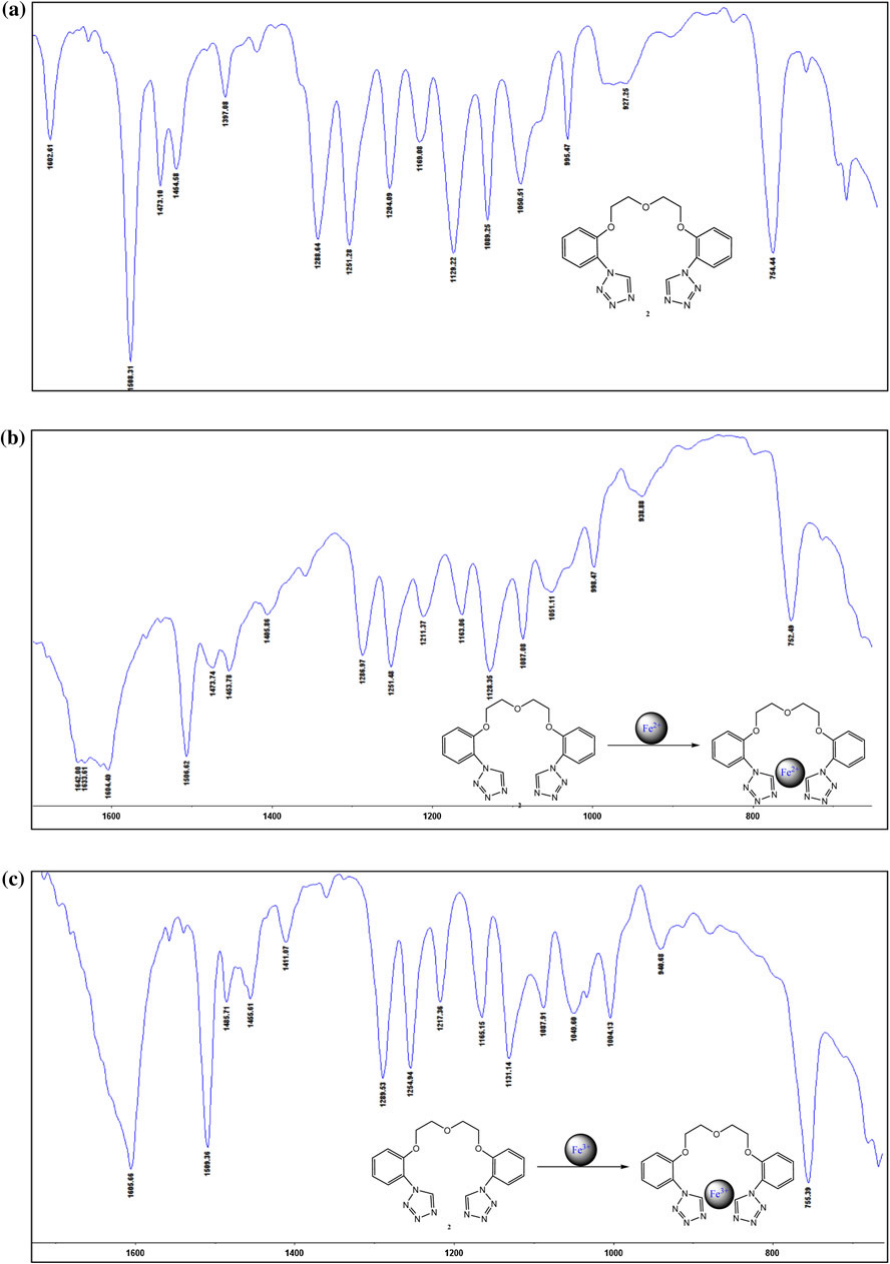
**a** Mid range FT-IR spectrum (film) of the ligand **2** **b** ligand with FeCl_2_ **c** ligand with FeCl_3_

In the preliminary experiments we investigate the spectroscopic characterization of the ligand **2** and its complex with ferrous(II) and ferrous(III) chloride. FT–IR Spectra of the ligand and the complexes have been taken within the range of 3500–500 cm^−1^. The spectra are depicted in Fig. 9. The range between 1700 and 500 cm^−1^ is particular interesting for us. The bands at 1508, 1473, 1454, 1129 cm^−1^ are due to combinations of bond stretching vibrations typical for coordinated tetrazole rings (Fig. 9a). The band at 1602 cm^−1^ is due to stretching vibration of the C=C bond from phenyl rings. The absorption of at 754 cm^−1^ is typical for bond angle deformation of the substitution tetrazole rings in the phenyl ring. After complexation up-shift of 40 cm^−1^ can be observed in the spectrum of the complexation attempt with iron(II) and small up-shift of 3 cm^−1^ for iron(III). The strong absorption at 1602 cm^−1^ is shifted to the 1642 cm^−1^. The coordination of the tetrazole N-atom to the iron(II) can be observed through strong force constants of the neighboring bonds from phenyl rings. No changes in the intensities of absorption characteristic for polyether chain can confirm our earlier hypothesis that it is not engaged in complex.

## Conclusion

A novel group of bis-tetrazole containing aromatic units and aliphatic chains with sulphur, nitrogen and oxygen atoms was synthesized. The bis-tetrazole was synthesized by using amines and ethyl orthoformate, sodium azide in glacial acetic acid. It was found that the yield of the products is polyether’s chains occurrence dependent. The X-ray structure of compound **2** was reported. Complexing properties of compounds **1**–**7** have been studied by spectrophotometric titration and IR.

### Experimental section

All materials and solvents were of analytical reagent grade. Thin layer chromatographies (TLC) were performed on aluminium plates covered with Silica gel 60 *F*_254_ (Merck). ^1^H NMR spectra were taken on Varian instrument at 200 and 500 MHz. IR spectra were recorded on Genesis II FTIR (Mattson) instrument. UV–Vis measurements were carried with the use of UNICAM UV 300 Series spectrophotometer. Elemental analyses were obtained on EAGER 200 apparatus. The melting points were uncorrected. All measurements were performed out at room temperature.

## Syntheses

The starting diamines: 1,2-bis(2-aminophenoxy)ethane, 1,5-bis(2-aminophenoxy)-3-oxapentane, 1,5-bis[2-aminophenyl(thio)]-3-oxapentane hydrochloride and 1,8-bis(2-aminophenoxy)-3,6-dioxaoctane were synthesized as described earlier in literature [13–16].

### General procedure for synthesis the bis-tetrazoles

A mixture of respective diamine (5 mmol), sodium azide (0.78 g, 12 mmol) and triethyl orthoformate (2.49 ml, 15 mmol) was dissolved in 10 ml glacial acetic acid and was stirred and heated at 90 °C for 9–10 h. It was subsequently cooled to room temperature and the resulting solution was poured into 50 ml of water and the precipitate was filtered off. The crude product (**1**–**5**) was purified by recrystallization using distilled water. Compounds **6** and **7** were purified by column chromatography on silica gel. As an eluent, methylene chloride (at the beginning) and then a mixture of methylene chloride–methanol (50:1) was used.

#### 1,2-Bis[2-(1*H*-tetrazol-1-yl)phenoxy]ethane (**1**)

Yield 99 % (1.73 g), white solid, mp 215–216 °C. ^1^H NMR (500 MHz, d-DMSO): δ = 4.35 (s, 4H, CH_2_), 7.04 (d, *J* = 8.31 Hz, 2H, Ar), 7.10 (m, 2H, Ar), 7.41 (m, 2H, Ar), 7.62 (d, *J* = 7.33 Hz, 2H, Ar), 8.95 (s, 2H, tetrazole) ppm. IR (KBr): 3137, 3090, 2962, 2900, 1510, 1489, 1473, 1397, 1288, 1252, 1171, 1133, 1085, 1036, 746 cm^−1^. Anal. calcd. for C_16_H_14_N_8_O_2_: C 54.85, H 4.03, N 31.98. Found: C 54.91, H 4.08, N 31.87.

#### 1,5-Bis[2-(1*H*-tetrazol-1-yl)phenoxy]-3-oxapentane (**2**)

Yield 98.5 % (1.94 g), white solid, mp 85–88 °C. ^1^H NMR (500 MHz, CDCl_3_): δ = 3.81 (t, *J* = 4.4 Hz, 4H, CH_2_), 4.27 (t, *J* = 4.39 Hz, 4H, CH_2_), 7.15 (m, 4H, Ar), 7.46 (m, 2H, Ar), 7.82 (d, *J* = 6.35 Hz, 2H, Ar), 9.34 (s, 2H, tetrazole) ppm. IR (KBr): 3127, 3069, 2952, 2891, 1512, 1473, 1453, 1399, 1297, 1253, 1175, 1142, 1132, 1084, 745 cm^−1^. Anal. calcd. for C_18_H_18_N_8_O_3_: C 54.82, H 4.60, N 28.41. Found: C 54.78, H 4.54, N 28.37.

#### 1,8-Bis[2-(1*H*-tetrazol-1-yl)phenoxy]-3,6-dioxaoctane (**3**)

Yield 97 % (2.12 g), white solid, mp 110–112 °C. ^1^H NMR (500 MHz, d-DMSO): δ = 3.71 (m, 6H, CH_2_), 4.25 (m, 6H, CH_2_), 7.16 (m, 2H, Ar), 7.32 (d, *J* = 7.69 Hz, 2H, Ar), 7.50 (m, 2H, Ar), 7.67 (d, *J* = 6.31 Hz, 2H, Ar), 9.71 (s, 2H, tetrazole) ppm. IR (KBr): 3150, 3073, 2948, 2888, 1507, 1470, 1448, 1394, 1294, 1256, 1166, 1131, 1100, 750 cm^−1^. Anal. calcd. for C_20_H_22_N_8_O_4_: C 54.79, H 5.06, N 25.56. Found: C 54.73, H 5.12, N 25.48.

#### 1,5-Bis[2-(1*H*-tetrazol-1-yl)phenoxy]-3-phenylazapentane (**4**)

Yield 90.6 % (0.25 g), brown solid, mp 146–148 °C. ^1^H NMR (500 MHz, d-DMSO): δ = 3.58 (m, 4H, CH_2_), 4.15 (m, 4H, CH_2_), 6.61 (m, 4H, Ar), 7.12 (m, 2H, Ar), 7.29 (d, *J* = 8.06 Hz, 2H, Ar), 7.55 (t, *J* = 7.69 Hz, 3H, Ar), 7.66 (d, *J* = 7.69 Hz, 2H, Ar), 9.77 (s, 2H, tetrazole) ppm. IR (KBr): 3147, 3081, 2935, 2883, 1599, 1510, 1473, 1453, 1289, 1251, 1165, 1128, 1088, 751 cm^−1^. Anal. calcd. for C_24_H_23_N_9_O_2_: C 61.39, H 4.94, N 26.85. Found: C 61.47, H 4.99, N 26.93.

#### 1,5-Bis[2-(1*H*-tetrazol-1-yl)phenoxy]-3-(4-toluenesulfonyl)azapentane (**5**)

Yield 46 % (0.075 g), brown solid, mp 103–108 °C. ^1^H NMR (500 MHz, d-DMSO): δ = 2.37 (s, 3H, CH_3_), 3.65 (m, 4H, CH_2_), 4.14 (m, 4H, CH_2_), 6.88 (m, 4H, Ar), 7.39 (d, *J* = 8.06 Hz, 2H, Ar), 7.76 (d, *J* = 8.06 Hz, 2H, Ar), 8.06 (m, 4H, Ar), 8.88 (s, 2H, tetrazole) ppm. IR (KBr): 3135, 3078, 2975, 2881, 1539, 1463, 1453, 1330, 1289, 1251, 1185, 1158, 1088, 1043, 970, 894, 740, 694, 650, 548 cm^−1^. Anal. calcd. for C_25_H_25_N_9_O_4_S: C 54.83, H 4.60, N 23.02, S 5.86. Found: C 54.79, H 4.68, N 23.13, S 5.91.

#### 1,5-Bis[2-(1*H*-tetrazol-1-yl)phenyl(thio)]-3-phenylazapentane (**6**)

Yield 45 % (0.45 g) of yellow oil. TLC (methylene chloride–methanol, 30:1). ^1^H NMR (500 MHz, CDCl_3_): δ = 2.86 (t, *J* = 6.84 Hz, 4H, CH_2_), 3.29 (t, *J* = 7.32 Hz, 4H, CH_2_), 7.17 (t, *J* = 7.32 Hz, 3H, Ar), 7.45 (m, 5H, Ar), 7.53 (m, 3H, Ar), 7.57 (d, *J* = 7.32 Hz, 2H, Ar), 8.90 (s, 2H, tetrazole) ppm. IR (film): 3129, 3096, 3065, 3010, 2927, 1693, 1596, 1496, 1462, 1356, 1279, 1200, 1052, 1045, 997, 874, 754 cm^−1^. Anal. calcd. for C_24_H_23_N_9_S_2_: C 57.46, H 4.62, N 25.13. Found: C 57.39, H 4.57, N 25.28.

#### 1,5-Bis[2-(1*H*-tetrazol-1-yl)phenyl(thio)]-3-oxapentane (**7**)

Yield 65 % (0.26 g) of yellow oil. ^1^H NMR (500 MHz, CDCl_3_): δ = 2.89 (t, *J* = 5.86 Hz, 4H, CH_2_), 3.41 (t, *J* = 6.35 Hz, 4H, CH_2_), 7.46 (m, 4H, Ar), 7.54 (t, *J* = 6.84 Hz, 2H, Ar), 7.65 (d, *J* = 7.82 Hz, 2H, Ar), 9.05 (s, 2H, tetrazole) ppm. IR (film): 3137, 3080, 2945, 2555, 1584, 1526, 1474, 1146, 1163, 1297, 1143, 1087, 1007, 761, 467 cm^−1^. Anal. calcd. for C_18_H_18_N_8_OS_2_: C 50.69, H 4.25, N 26.27, S 15.03. Found: C 50.73, H 4.32, N 26.19, S 15.14.

### Synthesis of substrates

#### 1,5-Di(4-toluenesulfonyloxy)-3-phenylazapentane

*N*-Phenyldiethanolamine (7.25 g, 40 mmol) was suspended in 100 ml pyridine and cooled with ice at 0 °C. To stirring mixture, three portions of 4-toluenesulfonyl chloride (30.64 g, 160 mmol) were added over a period of 30 min. The mixture was allowed to stay at 5 °C for 24 h. After ice addition, crude product was precipitated. Pure product was obtained by crystallized from petroleum ether. Yield 18.05 g (92 %) of white solid, mp 91–93 °C. ^1^H NMR (500 MHz, CDCl_3_): δ = 2.43 (s, 6H, CH_3_), 3.57 (t, *J* = 6.19 Hz, 4H, CH_2_), 4.11 (t, *J* = 6.02 Hz, 4H, CH_2_), 6.51 (d, *J* = 8.06 Hz, 2H, Ar), 6.74 (t, *J* = 7.32 Hz, 1H, Ar), 7.15 (t, *J* = 8.3 Hz, 1H, Ar), 7.26 (d, *J* = 7.61 Hz, 5H, Ar), 7.71 (d, *J* = 8.3 Hz, 4H, Ar) ppm. IR (KBr): 2958, 1925, 1598, 1505, 1579, 1230, 1150, 1096, 965, 889, 851, 819, 778, 747, 665, 554, 502 cm^−1^.

#### 1,5-Bis(2-nitrophenoxy)-3-phenylazapentane

A mixture of 2-nitrophenol (1.11 g, 8 mmol), 1,5-di(4-toluenesulfonyloxy)-3-phenylazapentane (1.95 g, 4 mmol) and anhydrous potassium carbonate (1.1 g) in *N,N*-dimethylformamide (5 ml) was heated at 100 °C for 8 h. After 8 h TLC (eluent, petroleum ether–diethyl ether, 1:2 v/v) confirmed that the starting material had completely been used. The mixture was then diluted with water; the precipitate was separated, washed with water, dried and next recrystallized from propan-2-ol. Yield 1.67 g (99 %) of pale yellow solid, mp 111–113 °C. ^1^H NMR (200 MHz, CDCl_3_): δ = 4.02 (t, *J* = 5.08 Hz, 4H, CH_2_), 4.31 (t, *J* = 5.06 Hz, 4H, CH_2_), 6.76 (m, 3H, Ar), 7.00 (m, 3H, Ar), 7.26 (t, *J* = 8.01 Hz, 3H, Ar), 7.48 (m, 2H, Ar), 7.82 (m, 2H, Ar) ppm. IR (KBr): 2930, 2882, 1608, 1510, 1347, 1272, 1252, 1195, 1168, 1038, 902, 849, 740, 694, 696, 611 cm^−1^.

#### 1,5-Bis(2-aminophenoxy)-3-phenylazapentane

1,5-Bis(2-nitrophenoxy)-3-phenylazapentane (0.42 g, 1 mmol) was suspended in 3.2 ml of ethanol/concentrated hydrochloric acid. To reaction mixture, SnCl_2_^.^2H_2_O (2.03 g, 9 mmol) in 2.5 ml concentrated hydrochloric acid was added dropwise over a period of 30 min. The mixture was refluxed for 8 h with reaction progress control by TLC (diethyl ether). After cooling the reaction mixture was diluted with water and extracted with methylene chloride. The extract was dried with MgSO_4_ and evaporated under reduced pressure. Pure product, beige–yellow oil, 0.26 g (71 %) was recrystallized from propan-2-ol. ^1^H NMR (200 MHz, CDCl_3_): δ = 3.91 (t, *J* = 5.86 Hz, 4H, CH_2_), 4.21 (t, *J* = 5.86 Hz, 4H, CH_2_), 6.73 (m, 6H, Ar), 6.80 (t, *J* = 7.32 Hz, 3H, Ar), 6.86 (d, *J* = 7.81 Hz, 2H, Ar), 7.26 (t, *J* = 7.31 Hz, 2H, Ar) ppm. IR (film): 3450, 2930, 2880, 1598, 1507, 1453, 1347, 1312, 1272, 1252, 1190, 1163, 1035, 900, 849, 740, 694, 696 cm^−1^.

#### 1,5-Di(4-toluenesulfonyloxy)-3-(4-toluenesulfonyl)azapentane

Diethanolamine (3.83 ml, 40 mmol) was suspended in 60 ml pyridine and cooled with ice at 0 °C. To stirring mixture, three portions of 4-toluenesulfonyl chloride (45.86 g, 240 mmol) were added over a period of 30 min. The mixture was allowed to stay at 5 °C for 24 h. After ice addition, crude product was precipitated. Pure product was obtained by crystallized from benzene. Yield 9.0 g (39.6 %) of green solid, mp 95–98 °C. ^1^H NMR (200 MHz, d-DMSO): δ = 2.37 (s, 3H, CH_3_), 2.42 (s, 6H, CH_3_), 3.28 (t, *J* = 5.85 Hz, 4H, CH_2_), 3.99 (t, *J* = 5.86 Hz, 4H, CH_2_), 7.35 (d, *J* = 7.81 Hz, 2H), 7.48 (d, *J* = 7.81 Hz, 4H), 7.56 (d, *J* = 8.3 Hz, 2H), 7.72 (d, *J* = 8.3 Hz, 4H) ppm. IR (KBr): 2953, 1936, 1597, 1494, 1453, 1357, 1307, 1197, 1097, 1089, 859, 815, 738, 654, 517 cm^−1^.

#### 1,5-Bis(2-nitrophenoxy)-3-(4-toluenesulfonyl)azapentane

A mixture of 2-nitrophenol (2.22 g, 16 mmol), 1,5-di(4-toluenesulfonyloxy)-3-(4-toluenesulfonyl)azapentane (4.5 g, 8 mmol) and anhydrous potassium carbonate (2.21 g) in dimethylformamide (10 ml) was heated at 100 °C for 8 h. The mixture was then diluted with water; the precipitate was separated, washed with water, dried and next recrystallized from propan-2-ol. Yield 3.96 g (99 %) of pale beige solid, mp 117–119 °C. ^1^H NMR (200 MHz, CDCl_3_): δ = 2.31 (s, 3H, CH_3_), 3.87 (t, *J* = 5.37 Hz, 4H, CH_2_), 4.31 (t, *J* = 5.37 Hz, 4H), 7.02 (m, 4H), 7.15 (d, *J* = 7.81 Hz, 2H), 7.52 (t, *J* = 8.3 Hz, 2H), 7.68 (d, *J* = 8.3 Hz, 2H), 7.82 (d, *J* = 6.35 Hz, 2 H) ppm. IR (KBr): 2966, 2882, 1608, 1523, 1486, 1356, 1335, 1280, 1257, 1156, 1088, 1048, 1014, 967, 895, 748, 716, 696, 641, 547 cm^−1^.

#### 1,5-Bis(2-aminophenoxy)-3-(4-toluenesulfonyl)azapentane

1,5-Bis(2-nitrophenoxy)-3-(4-toluenosulfonylo)azapentan (0.25 g, 0.5 mmol) was suspended in 1.6 ml of ethanol/concentrated hydrochloric acid. To reaction mixture, SnCl_2_·2H_2_O (1 g, 4.5 mmol) in 1.25 ml concentrated hydrochloric acid was added dropwise over a period of 30 min. The mixture was refluxed for 6 h with reaction progress control by TLC (methylene chloride–methanol, 10:1). After cooling the reaction mixture was diluted with water and extracted with methylene chloride. The extract was dried with MgSO_4_ and evaporated under reduced pressure. Pure product, beige–yellow oil, 0.176 g (71 %) was recrystallized from propan-2-ol. ^1^H NMR (200 MHz, CDCl_3_): δ = 2.40 (s, 3H, CH_3_), 3.70 (t, *J* = 5.37 Hz, 4H, CH_2_), 4.14 (t, *J* = 5.37 Hz, 4H, CH_2_), 6.64 (m, 2H, Ar), 6.86 (m, 6H, Ar), 7.35 (d, *J* = 7.32 Hz, 2H, Ar), 7.26 (d, *J* = 7.81 Hz, 2H, Ar) ppm. IR (film): 3434, 2976, 2880, 1618, 1527, 1453, 1335, 1322, 1287, 1255, 1187, 1159, 1085, 1044, 970, 894, 740, 694, 656, 550 cm^−1^.

#### 1,5-Bis[2-aminophenyl(thio)]-3-phenylazapentane hydrochlochloride

2-Aminothiophenol (1.07 ml, 10 mmol), 1,5-di(4-toluenesulfonyl)oxy-3-phenylazapentane (2.44 g, 5 mmol) and anhydrous potassium carbonate (1.38 g) in dimethylformamide (10 ml) were heated for 24 h at 100 °C. The solvent was removed under reduced pressure and the crude product was diluted with water and extracted with methylene chloride. The extract was dried with MgSO_4_ and evaporated under reduced pressure. Bisamine was then dissolved in methanol and concentrated hydrochloric acid (0.32 ml) to provide red solid of 1,5-bis[2-aminophenyl(thio)]-3-phenylazapentane hydrochloride (2.32 g, 98 %), mp 168–171 °C. ^1^H NMR (500 MHz, d-MeOH): δ = 3.11 (m, 4H, CH_2_), 3.68 (m, 4H, CH_2_), 7.02 (m, 3H, Ar), 7.31 (t, *J* = 7.63 Hz, 3H, Ar), 7.43 (m, 5H, Ar), 7.66 (d, *J* = 7.63 Hz, 2H, Ar) ppm. IR (KBr): 3420, 2827, 2580, 2547, 1972, 1599, 1555, 1503, 1494, 1444, 1280, 1214, 1182, 1141, 759, 691, 452 cm^−1^.

#### 1,5-Bis[2-aminophenyl(thio)]-3-oxapentane hydrochloride

A mixture of 2-aminothiophenol (2.14 ml, 20 mmol), 1,5-dichloro-3-oxapentane (1.17 ml, 10 mmol) and anhydrous potassium carbonate (2.76 g) in 10 ml of dimethylformamide was heated for 24 h at 80 °C. The reaction progress was monitored by TLC using mixture of petroleum ether–ethyl acetate (4:1 v/v) as a mobile phase. The reaction mixture was cooled to room temperature and extracted with methylene chloride (2 × 20 ml) and water (20 ml). The organic phase was dried over MgSO_4_ and concentrated under reduced pressure. Pure product was obtained with the use of column chromatography with the same mixture of solvents as TLC. The product was subsequently dissolved in methanol and concentrated hydrochloric acid to give white solid of 1,5-bis(2-aminophenylsulfide)-3-oxapentane hydrochloride, mp 197–199 °C. Yield 2.75 g (70 %). ^1^H NMR (200 MHz, CDCl_3_): δ = 2.9 (t, *J* = 6.0 Hz, 4H, CH_2_), 3.41 (t, *J* = 6.05 Hz, 4H, CH_2_), 6.18–7.12 (m, 8H, Ar). IR (KRr): 3437, 2900, 2560, 1992, 1584, 1556, 1526, 1474, 1446, 1297, 1097, 1007, 757, 467 cm^−1^. The synthesis was performed analogously to the synthesis compounds described in literature.

#### Spectrophotometric studies of metal cation complexation

UV–Vis titration was carried out by addition of metal chloride to the bis-tetrazole solution. Titrations were carried out in 1 cm path length quartz cuvette keeping constant volume of the ligand solution (2 ml). Titration step 0.01 ml.

## Supplementary material

Complete crystallographic data of structure have been deposited with the Cambridge Crystallographic Data Centre, CCDC Nos. 859174. Copies of this information may be obtained free of charge from The Director, CCDC, 12 Union Road, Cambridge CB2 1EZ, UK (fax: +44-1223-3336-033, e-mail:deposit@ccdc.cam.ac.uk).
